# Denoising PCR-amplified metagenome data

**DOI:** 10.1186/1471-2105-13-283

**Published:** 2012-10-31

**Authors:** Michael J Rosen, Benjamin J Callahan, Daniel S Fisher, Susan P Holmes

**Affiliations:** 1 Department of Applied Physics, Stanford University, CA, US; 2 Departments of Biology and Bioengineering, Stanford University, CA, US; 3 Department of Statistics, Stanford University, CA, US

## Abstract

**Background:**

PCR amplification and high-throughput sequencing theoretically enable the characterization of the finest-scale diversity in natural microbial and viral populations, but each of these methods introduces random errors that are difficult to distinguish from genuine biological diversity. Several approaches have been proposed to denoise these data but lack either speed or accuracy.

**Results:**

We introduce a new denoising algorithm that we call *DADA* (Divisive Amplicon Denoising Algorithm). Without training data, *DADA* infers both the sample genotypes and error parameters that produced a metagenome data set. We demonstrate performance on control data sequenced on Roche’s *454* platform, and compare the results to the most accurate denoising software currently available, *AmpliconNoise*.

**Conclusions:**

*DADA* is more accurate and over an order of magnitude faster than *AmpliconNoise*. It eliminates the need for training data to establish error parameters, fully utilizes sequence-abundance information, and enables inclusion of context-dependent PCR error rates. It should be readily extensible to other sequencing platforms such as *Illumina*.

## Background

The potential of high-throughput sequencing as a tool for exploring biological diversity is great, but so too are the challenges that arise in its analysis. These technologies have made possible the characterization of very rare genotypes in heterogeneous populations of DNA at low cost. But when applied to a metagenomic sample, the resulting raw data consist of an unknown mixture of genotypes that are convolved with errors introduced during amplification and sequencing.

There are two broad approaches to high-throughput sequencing of metagenomes: in *amplicon sequencing* (also called *gene-centric* or *gene-targeted* metagenomics) a pool of DNA for sequencing is produced by using PCR to amplify all the variant sequences in a sample that begin and end with a chosen pair of primers [1-3], frequently targeting hypervariable regions of the 16S ribosomal RNA gene [4]; in *de novo genome assembly* total DNA is sequenced without amplification and reads are clustered into “species bins”, each providing the material for genome assembly by shotgun methods (see Table 1 in [5] for a list of such studies).

By trading off a broad survey of gene content for greater sequencing depth at the sampled loci, amplicon sequencing has the potential to detect the rarest members of the sampled community, but errors interfere more profoundly. Unlike genome assembly projects, where one needs only to determine the consensus base at each locus or decide whether a SNP is present in a population, the space of possible distributions for the sample genotypes and frequencies is effectively infinite. As a result, ambiguities in genome projects can usually be resolved by increasing the amount of data, whereas increasing depth (as much as 10^6^ in recent studies [6,7]) increases the number of both real and error-containing sequences and makes the challenge of distinguishing minority variants from errors only greater under amplicon sequencing. Greater depth therefore calls for progressively more sophisticated methods of analysis.

The analysis of amplicon sequence data typically begins with the construction of OTUs (operational taxonomic units), clusters of sequences that are within a cutoff in Hamming distance from one another. OTUs serve to collapse the complete set of sequences into a smaller collection of representative sequences – one for each OTU – and corresponding abundances based on the number of reads falling within each cluster. OTUs were developed as a tool for classifying microbial species, but have also been repurposed to the task of correcting errors; the sequences within an OTU are typically interpreted as a taxonomic grouping without specifying whether the variation within an OTU represents errors or real diversity on a finer scale than that chosen to define the OTU. If the scale of the noise is smaller than that of the clusters, then the construction of OTUs will appropriately group error-containing sequences together with their true genotype. However, as sequencing depth increases, low probability errors outside the OTU radius will start to appear, and will be incorrectly assigned to their own OTU. Early studies using this approach on high-throughput metagenome data sets reported large numbers of low-abundance, previously unobserved genotypes that were collectively dubbed the *rare biosphere*[8]. Later, analyses of control data sets indicated that the diversity estimates in such studies tends to be highly inflated [9] and that results may lack reproducibility [10]. The dual purpose of OTUs for correcting errors and for taxonomic grouping is appropriate when the diversity is being sampled at a coarse level, e.g. the frequency of different phlya. However, when probing finer-scale diversity, OTU methods have intrinsically high false positive and false negative rates: they both overestimate diversity when there exist errors larger than the OTU-defining cutoff and cannot resolve real diversity at a scale finer than that (arbitrary) cutoff.

In response, a variety of approaches to disentangling errors from actual genetic variation have been proposed recently [11-14]. These include multiple rounds of OTU clustering with different hierarchical methods [11], utilizing sequence abundance information implicitly by starting new clusters with common sequences [11,12], and replacing OTU clustering with an *Expectation-Maximization* (EM) approach [13,14]. Accuracy has steadily improved, but all methods still fall short of maximizing the information acquired from metagenome data sets.

We believe that the way forward is to model the error process and evaluate the validity of individual sequences in the context of the full metagenomic data set, crucially including the abundances (number of reads) corresponding to each sequence. Major progress in this direction has been made recently by *Quince et al*[13,14]. In the specific context of pyrosequencing, often used for metagenomics, strings of the same nucleotide (homopolymers) are problematic, and *Quince et al* incorporated a model of the distribution of homopolymer light intensities into an *Expectation-Maximization* (EM) algorithm, *Pyronoise*, which infers the homopolymer lengths of sequencing reads [13] (*Q09*). Later, *Quince et al* released *AmpliconNoise*, an extension of *PyroNoise* that includes rates of single-nucleotide substitution errors obtained from training data [14] (*Q11*). These methods were shown to more accurately infer the underlying sample genotypes than other approaches, demonstrating the worth of explicitly modeling errors. However, the methods of Quince et al. have several shortcomings that we would like to rectify: (i) as the size of sequence data sets grows, *AmpliconNoise* becomes too slow to use in many applications; (ii) estimation of error rates relies on the existence of training data specific to the PCR and sequencing chemistries used; (iii) differentiation of fine-scale diversity is limited because read abundances are not fully utilized when calculating the distance between sequences and clusters; (iv) the parameters that determine how conservative the algorithm is in inferring real diversity are ad hoc and cannot be tuned without experiment-specific training data.

We build on the error-modeling approach pioneered in *AmpliconNoise* by developing a novel algorithm, *DADA*, to denoise metagenomic amplicon sequence data that addresses the concerns raised above [15]. We start with a parametric statistical model of substitution errors. We incorporate this error model into a divisive hierarchical clustering algorithm that groups error-containing reads into clusters consistent with being derived from a single sample genotype. Finally we couple this clustering algorithm with the inference of the error parameters from the clustered data, and perform each step in alternation until both converge. This method is presented below, and is shown to outperform previous methods in both speed and accuracy on several control data sets.

## Results

### Model and algorithm

We introduce a first-order model of the error process by assuming (1) each sequence read originates from a distinct DNA molecule in the sample, and therefore that the presence of errors on *different reads* are statistically independent, and (2) errors on different sites of the *same read* are also statistically independent events. The independence of errors across different reads relies on the independence of the PCR replication histories of those reads, a condition that holds when the total number of reads is significantly smaller than the total number of DNA molecules present in the initial environmental sample and there are no strong amplification biases for sequences with errors.

Under these conditions, the numbers of reads (abundances) of the error-containing sequences derived from a sample genotype follow the multinomial distribution, and the abundance *r* of each particular sequence is binomially distributed (see Methods) with a probability *λ*determined by the *particular* combination of errors in that sequence and a number of trials *ρ* given by the total number of reads of its sample genotype. These facts allow us to establish two statistics to evaluate the hypothesis that a collection of sequencing reads derives from a single sample genotype. The *abundance p-value* determines when there are *too many reads* of the same sequence to be consistent with the error model, and the *read p-value* determines when a sequencing read is *too far away* to be an error from an inferred sample genotype.

These statistics serve as the basis of a sequence-clustering algorithm in which (1) reads are assigned to clusters, (2) a putative sample genotype is inferred for each cluster, (3) reads are reassigned to the cluster for which they are most likely to have resulted as errors from the inferred sample genotype, (4) the two p-value statistics are computed given the inferred sample genotypes and the clustering of the sequences (5) additional clusters are created if the clustering is statistically inconsistent with the error model (as suggested by small p-values).

**Figure 1:**
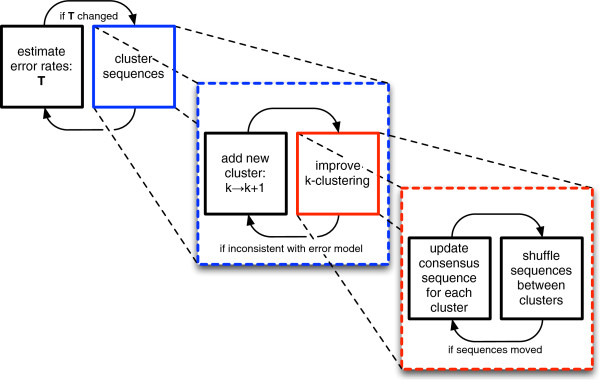
***DADA schematic.*** The basic structure of *DADA*, an algorithm to denoise amplicon sequence data. See Algorithm Algorithm 1 in the Methods section for the pseudocode and a more detailed description.

The full algorithm (Figure 1) combines this probabilistic sequence clustering with the estimation of substitution-error probabilities that are used to compute the p-values. The algorithm begins by assuming all reads derive from a single sample genotype and estimates initial error probabilities given this assumption. It then alternates between clustering the reads and re-estimating the error probabilities until it converges to a final set of mutually consistent clusters and error probabilities.

#### The p-values

We introduce two statistics for deciding that particular sequences *did not* arise from errors. The *read p-value* is the probability of having observed at least one read of a sequence that is as improbable as the most improbable sequence amongst the observed reads. This statistic treats each *read* as a separate event (giving rise to its name) and therefore does not utilize sequence abundance. It results in a hard cutoff, *λ*^∗^, below which reads are decided not to be errors by *DADA*. This cutoff is set by the choice of a significance threshold *Ω*_*r*_, the probability of having observed at least one read more unlikely than *λ*^∗^. The *abundance p-value*, which is computed for each sequence individually, is the probability of having observed at least as many identical reads as we did of each sequence (conditioned on having observed at least one). The conservativeness of this measure is set by a significance threshold *Ω*_*a*_, the probability that at least one sequence should have been as overabundant as the most overabundant sequence. The abundance p-value gives *DADA* significantly greater sensitivity than previous methods.

**Figure 2:**
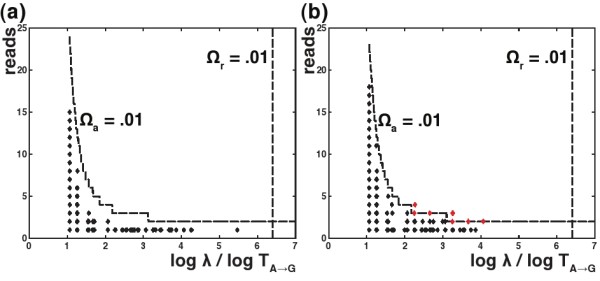
**Discrimination plots for a typical cluster in the *Artificial *data set with 4691 reads. (a)** simulated errors drawn from the error model and **(b)** the real errors in the cluster. Sequences (diamonds) are characterized by abundance and the probability *λ*per read of having been produced. On the x-axis, we plot log*λ*scaled by the most common error probability, *T*_A→G_, so that values can be interpreted as an *effective* Hamming distance. The dashed lines delineate the region – the lower left quadrant – where, for significance thresholds *Ω*_*a*_and *Ω*_*r*_ provided by the user, *DADA* accepts that a sequence could have arisen via the error model. The vertical dashed lines shows the *λ*below which (or the effective distance above which) the read p-value rejects sequences as being errors, and the curved dashed line shows the abundances above which the abundance p-value rejects sequences as being errors for each value of *λ*. There are several sequences in the real data (red diamonds) that would be rejected by the abundance p-value at the *Ω*_*a*_ = .01 significance level; we posit that early round PCR effects are a suitable candidate to explain these departures from the error model.

Figure 2 shows simulated and real data from a typical cluster of sequences that originated from a common genotype (from the *Artificial* data set, introduced below). The abundance *r* and probability *λ* of each sequence is plotted, as the ability of the read and abundance p-values to discriminate between errors and non-errors is easily visualized in this parameter space. The regions where *DADA* will declare a sequence to be an error or a real sequence are delineated by a dashed line for each of our p-value statistics. The *λ* values have been log-transformed and scaled by the most common error probability, making the x-axis interpretable as an effective Hamming distance. Due to this scaling, it is also useful to interpret this plot in terms of real Hamming distances, in which case the *Ω*_*a*_line represents a lower bound on *DADA*’s resolution for *any* error at that distance.

For both the real and simulated data, the abundance p-value does a good job of tracking the form of the abundances of the errors, and the read p-value sits to the right of all observed data. For the real data, a small number of errors sit on or above the abundance discrimination line. Such errors were individually not expected to be observed at all, but ended up with a small number of reads larger than one. This pattern was observed across many clusters, and we believe that it reveals the presence of small violations of our assumption of the independence between reads. In particular, in a regime where the ratio of the number of error-free reads to the number of DNA molecules in the sample that act as the basis for amplification is of order one or larger, then errors during early stages of PCR may be sampled multiple times in the sequence data. As a result, the distribution for the number of reads of these errors may fall off much more slowly than what our model suggests. To deal with this effect in this paper, we lowered the *Ω*_*a*_threshold using an ad hoc method (discussed below) to prevent excess false positives. Doing so did not affect *DADA*’s ability to detect the genuine diversity in the data analyzed in this paper, which was typical of the data analyzed in many microbial metagenomics studies, but the sensitivity that is lost by using very small values of *Ω*_*a*_ could be limiting for samples with even finer-scale diversity. Further analytics that model PCR as a branching process improve this current ad hoc threshold (unpublished work).

#### Treatment of insertions and deletions

*DADA* does not attempt to explicitly model the indel error processes, and indels do not contribute to the determination of whether sequences are related to each other via errors. Instead, sequences are aligned to each putative sample genotype, and are assigned to clusters on the basis of substitutions. During the computation of p-values, we sum together the reads of sequences within each cluster that have the same set of substitutions (forming structures that we call *indel families*). The number of reads of each of these indel families, rather than those of the raw sequences, are the basis of our p-values (see Methods).

Treating indels in this way does not affect the accuracy of *DADA* for the test data sets analyzed here, as the sample genotypes all differed from each other by at least one substitution, and these provided enough information for *DADA* to distinguish between them. However, *DADA* cannot distinguish between sequences that differ *only* by indels. In such cases, if the amplicons being denoised are coding regions, frame information should be used for making decisions about whether particular indels are real or errors, but in order to denoise non-coding regions with pure indel diversity, *DADA* is not sufficient in its current form.

#### Preclustering

Prior to our probabilistic sequence clustering we divided the raw data into coarse 3% single-linkage clusters (with indels not contributing to distance), subsets for which each sequence is ≤3*%* from at least one other sequence in its cluster and >3*%*from all sequences in other clusters. Due to its speed, we employed the *ESPRIT* algorithm for this task [16]. Single-linkage’s propensity for *chaining* was advantageous in this circumstance, as all error-containing sequences are very likely to be in the same cluster as their originating sample genotypes; for a sample genotype and one of its errors to end up in different clusters, the error would have to be ≥3*%* from the nearest *error* clustered with that genotype, corresponding to a large gap in the error cloud, which is unlikely under our error model.

#### Clustering

Each precluster is partitioned into sets of sequences that are conjectured to contain all errors arising from different sample genotypes. This partition is initialized to a single cluster containing all sequences. Two procedures then alternate. First, the indel family most unlikely to have resulted from errors is split off into a new cluster. Sequences then move between clusters based on the probability that they were generated as errors by each one, and the consensus sequence for each cluster is updated until there are no remaining reassignments that can improve the probability of the data. This second step is analogous to the assignment and update steps of standard *k-means* clustering. This alternation stops when the partition of the sequences fits with the current error model at the significance levels provided by the user.

### Accuracy

We evaluated the accuracy of *DADA* by denoising three of the data sets in *Q11* used to demonstrate *AmpliconNoise*’s accuracy relative to the earlier *SLP* and *DeNoiser* algorithms. These data are derived from mixtures of known clones that were amplified together and sequenced on the *454* platform, and consisted of different hypervariable regions of the 16S RNA subunit of bacterial ribosomes (16S rRNA), which are commonly used as a proxy for phylogenetic diversity in metagenomic studies [4]. Two of the data sets, *Divergent* and *Artificial*, with 35,190 and 31,867 reads, were sequenced with the GS-FLX chemistry and were truncated at 220 nucleotides. They were constructed by amplifying the V5 region of the 16S rRNA gene from 23 and 90 clones, respectively, isolated from lake water. The *Divergent* clones were mixed in equal proportions and are separated from each other by a minimum nucleotide divergence of 7%, while the *Artificial* clones were mixed in abundances that span several orders of magnitude, with some of the clones differing by a single SNP. The other data set, *Titanium*, with 25,438 reads, was sequenced with the newer Titanium chemistry and was truncated at 400 nucleotides. It contains V4-5 16S rRNA genes from 89 clones isolated from Arctic soil with varying abundance and genetic distances, similar to the *Artificial* set.

All data sets had undergone filtering of reads deemed to be of low quality prior to application of *AmpliconNoise* in *Q11*, so for purposes of comparison, we denoised the same set of filtered reads. The presence of a small number of low-quality reads in 454 data has been previously demonstrated [17], and as we do not expect these to be well described by our error model, we encourage the use of such quality filtering before applying *DADA* to non-test data. As *SLP* and *DeNoiser* were already demonstrated to be less accurate than *AmpliconNoise* on these data, we include here *DADA*’s performance only relative to that of *AmpliconNoise*. There were six other data sets presented in *Q11* of V2 regions from a gut microbial community, but these had such an overwhelming number of chimeric sequences (reported to be as high as 85% in *Q11*), which neither *DADA* nor *AmpliconNoise* attempts to address, that we opted not to include these data sets in our analysis.

#### Tuning algorithmic sensitivity

*DADA* employs two tunable parameters that determine how conservative or liberal the algorithm is to be in deciding whether particular sequences could have resulted from errors: *Ω*_*a*_, and *Ω*_*r*_, the significance levels for its abundance and read p-values. Decisions about singletons, the sequences represented by a single read, depend on *Ω*_*r*_, whereas decisions about sequences with several reads depend on *Ω*_*a*_. The two values may be tuned independently to match the priority being placed on capturing the rarest and more common diversity.

**Figure 3:**
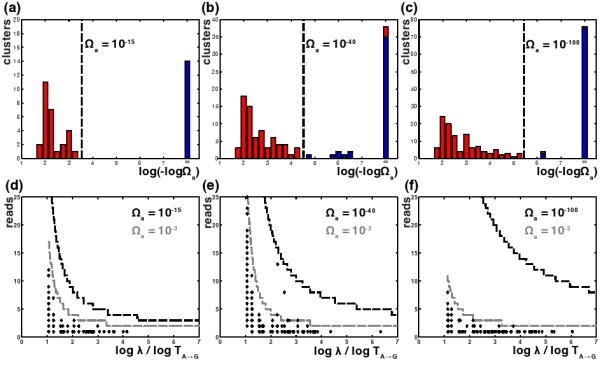
**Ad hoc ***Ω*_*a *_**choices for the *Divergent *(a) and (d), *Artificial *(b) and (e), and *Titanium *(c) and (f) data sets. (a)**-**(c)** are histograms of the *Ω*_*a*_threshold at which each cluster derived from a run of *DADA* with *Ω*_*a*_=*Ω*_*r*_=10^−3^rejoins some other nearby cluster. Genuine genotype counts are shown in blue and false positive counts are shown in red. The first gaps in these histograms were used to pick *Ω*_*a*_thresholds for reclustering the data, and are indicated by vertical dashed lines. **(d)**-**(f)** show the *Ω*_*a*_discrimination lines for the largest cluster in each data set (with 2294, 5479, and 1095 reads) for *Ω*_*a*_=10^−3^and the associated ad hoc *Ω*_*a*_values.

Due to early-stage PCR effects discussed above, it was necessary to use *Ω*_*a*_ significance levels lower than typical values. In order to select such values, we first performed a loose clustering of each data set with larger values of *Ω*_*a*_ and *Ω*_*r*_ and then made histograms of the *Ω*_*a*_thresholds that would be required for each cluster to be reabsorbed into some other cluster (Figure 3). If there are errors with moderate statistical deviations from our model, we expect that these will show up as a tail of increasingly small p-values that will disappear smoothly as we lower the *Ω*_*a*_ threshold. Thus, we looked for the first large gap in these histograms that would suggest all such model departures had been captured. Such a gap occurs at *Ω*_*a*_=10^−15^ for the *Divergent* data, *Ω*_*a*_=10^−40^ for the *Artificial* data, and *Ω*_*a*_=10^−100^for the *Titanium* data. We used these values in the analysis that follows, but also clustered all three data sets with *Ω*_*a*_=10^−100^and found that the results were unchanged (see appendix Appendix 2: *Ω*_*a*_ robustness). This suggests that *Ω*_*a*_=10^−100^is a reasonable default value to use when clustering diversity at this scale, even though higher resolution may be achieved by the method outlined above. For non-test metagenome data that is more diverse and less oversampled, we have seen evidence that using much larger values of *Ω*_*a*_(such as .01) may be possible without compromised accuracy, but in such cases it is always advisable to make histograms of the type above to ensure that there is not an excess of clusters that would vanish if *Ω*_*a*_ were lowered slightly.

We did not observe any significant departures in these data from our model that would affect the read p-values, and it was therefore possible to maintain the interpretation of *Ω*_*r*_as a significance threshold. As a result, for these data, which contain ≤50 preclusters that were clustered separately by *DADA*, we set *Ω*_*r*_=10^−3^ so that the probability of having a false positive would be ≤5*%*for each data set.

#### False negatives and false positives

The purpose of *DADA* (and *AmpliconNoise*) is the inference of the genotypes present in the underlying sample from a set of noisy (error-containing) sequencing reads. There are two types of errors that such an algorithm can make: false positives in which a sample genotype is inferred that was not present in the sample, and false negatives in which the algorithm fails to infer a sample genotype that was present in the sequencing reads. The tradeoff between false positives and false negatives in the two algorithms can be controlled by the algorithmic parameters, depending on which type of error presents more of a problem to the user.

We present, in Table 1, a comparison of the false positives and false negatives for *DADA* and *AmpliconNoise* applied to the control data sets described above. Note, however, one important detail: these algorithms are designed to remediate substitution and indel errors, not all possible errors. In particular, we found that contaminants, chimeras, and pathological homopolymer errors contributed to these sequencing data sets. Using ad hoc methods, discussed in appendix Appendix 1: chimeras, contaminants, and missing or incorrect Sanger sequences, we accounted for these additional error sources, and did not penalize either algorithm for them.

**Table 1:** False positives and false negatives

	**DADA**		**AmpliconNoise**		
**Sample**	**False Pos**	**False Neg**	**False Pos**	**False Neg**
Divergent	0	0	2	0
Artificial	1	2	8	7
Titanium (s10)	6	0	8	9
Titanium (s25)			23	4

*DADA* is more accurate in its inference of the sample genotypes than is *AmpliconNoise* on every data set. The difference is especially strong among false negatives, where *DADA* successfully identifies virtually all sample genotypes; *DADA*’s only two false negatives, both in the *Artificial* set, result from pathological alignment issues between sequences that differ only in the last two bases.

**Figure 4:**
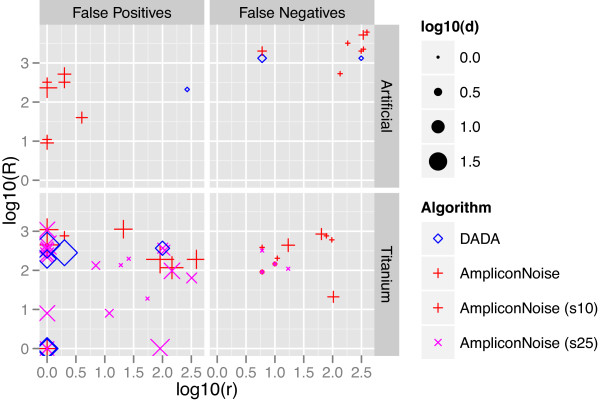
**Nature of false positives and false negatives of *DADA *and *AmpliconNoise *on *Artificial *and *Titanium *data sets.** False positives are characterized by the number of reads associated with the falsely inferred genotype *r*, the distance to the nearest real sequence *d*, and the number of reads associated with that nearest real sequence *R*. False negatives are characterized by the number of reads that matched the missing genotype *r*, the distance from that missing genotype to the nearest inferred genotype *d*, and the number of reads associated with that nearest inferred genotype *R*.

The differences in the nature of the false positives and negatives made by *DADA* and *AmpliconNoise* are shown in Figure 4. *DADA* produces one false positive in the *Artificial* data: a sequence with 268 reads that is a single substitution from a sample genotype with only 210 reads. Due to its vast abundance, this is unlikely to be an error, and we speculate it may represent a polymorphism that arose early in the growth of this clone. *AmpliconNoise* produces eight false positives in the *Artificial* data: all sequences with 1-4 reads that are (except for one) 1-4 substitutions away from clusters with a few hundreds of reads. Although these sequences were not atypical errors as judged by *DADA* due to their low abundances, *AmpliconNoise* calls them real as a result of setting a narrow error radius that is needed to prevent additional false negatives. The differences between the errors made by the two algorithms is less clear in the *Titanium* data set, but *DADA* outperforms *AmpliconNoise* in both FPs and FNs. We included *AmpliconNoise*’s results for the *Titanium* data set for both parameter settings included in *Q11*: the *σ*=.04 clustering (s25), which produces only four false negatives, leads to many false positives similar in nature to those of the *Artificial* clustering – low abundances and a small number of substitutions away from large clusters; the *σ*=.1 (s10) clustering produces many fewer false positives but misses nine sample genotypes.

### Speed

We evaluated the speed of *DADA* applied to the *Artificial* data, which was used to profile *AmpliconNoise* in *Q11* (Table 2). *ESPRIT* was run on a single core of an AMD Phenom II 3.2GHz running Ubuntu and *DADA* was run on a MacBook Pro with an Intel Core 2 Duo 2.4 GHz.

**Table 2:** CPU times for clustering *artificial community*

**Function**	**CPU time (seconds)**
ESPRIT	
kmerdist	74.80
needledist	924.68
Total	1002.68
DADA	
N-W alignments	97.81
read p-values	58.84
Total	296.64
ESPRIT+DADA	1.30×10^3^
AmpliconNoise	7.57×10^4^

The CPU times for a few significant subroutines within *DADA* and *ESPRIT* as well as their total CPU times.

*DADA* is currently written in MATLAB, but sequence alignments and the construction of *indel families* were bottlenecks that we reimplemented as MEX (Matlab executable) C programs. The majority of the time to run our denoising pipeline on the *Artificial* data set is spent on *ESPRIT*’s performance of pairwise alignments during the single-linkage pre-clustering step (*needledist*). A newer version of *ESPRIT* promises to be released soon that may dramatically lower this time [18]. If additional speedups are needed as data sets grow, it should be possible to replace the global alignments of *ESPRIT* by banded alignments that would be guaranteed to produce the same clusters if the width of the band is equal to the cluster radius, and would have have roughly linear (in sequence length) rather than quadratic, running time. Nonetheless, for these data *DADA* already gives a 60-fold speedup over *AmpliconNoise*, making jobs that required a 64-core cluster to run *AmpliconNoise* appropriate for a laptop running *DADA*.

As read lengths continue to grow, we expect the time complexity of *DADA* to be affected in two primary ways. First, because the complexity of the Needleman-Wunsch alignment algorithm used by both *DADA* and *ESPRIT* scales with the product of the lengths of the input sequences [19] there will be a quadratic slowdown with increasing read length unless heuristics are employed. On data sets comparable to those analyzed here, alignments consume the majority of algorithmic time and this scaling will dominate in the near future. Second, our current implementation for computing read p-values has both time and space complexity that grows very rapidly with read length (it is asymptotically $O(L^{11})$). This was not strongly limiting for these data, but in case it should become so as reads become longer, we have explored the use of a continuous approximation for the error probabilities that may alleviate this problem.

### PCR substitution probabilities: symmetries and nearest-neighbor context-dependence

**Figure 5:**
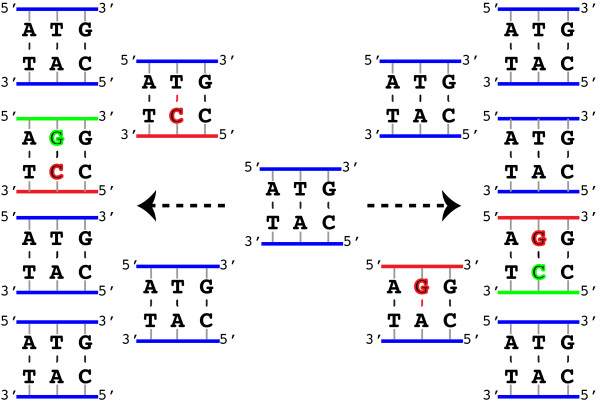
**Two paths to the same error.** Different mispaired bases (red) produce the same double stranded product once paired with complementary bases (green) so that each path leads to an ATG→AGG substitution error on one strand and a CAT→CCT on the other. The probability of these two errors is therefore expected to be very similar.

*DADA* not only infers sample genotypes, it also infers the substitution error probabilities caused by the amplification and sequencing processes. The substitution error probabilities inferred by *DADA* for all three data sets exhibit an approximate symmetry under complementation of the two bases involved. For example, the A→G probability is close to the T→C probability. This symmetry is expected if substitution errors predominantly arise during PCR amplification because substitution errors during PCR can be the result of either of two different mis-pairing events (from when the sequence is being copied to the opposite strand, or from when it is being copied back), and complementary substitution errors share causal mis-pairing events (see Figure 5 for a schematic). As it was not imposed, and the identities of the original genotypes were not known to the algorithm, the symmetry is a highly non-trivial check on *DADA*’s ability to learn error probabilities without training data. Additionally, the inferred substitution probabilities were similar across the data sets, and especially so between *Divergent* and *Artificial*, which were generated with the same PCR protocols.

**Figure 6:**
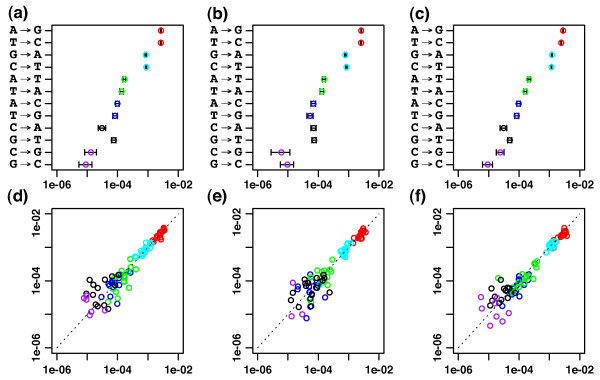
**Error probability symmetries for *Divergent *(a) and (d), *Artificial *(b) and (e), and *Titanium *(c) and (f) data sets. (a)**-**(c)**: context-independent substitution error probabilities inferred by *DADA* with 95% confidence intervals based on binomial sampling error. Note the approximate symmetry between *i*→*j*and $ī\to \bar{j}$ probabilities (which show up contiguously along the y-axis), where ī denotes the complement of nucleotide *i*. **(d)**-**(f)**: All 96 reverse-complementary pairs of context-dependent error probabilities inferred by *DADA* for each data set. For each pair, the probability of the error away from an A or C is plotted on the x-axis and the error probability away from T or G is plotted on the y-axis. The pairing between these probabilities – seen by the tendency to lie along the diagonal – is stronger for the largest probabilities, which have the least sampling noise. The colors signify complementary pairs of errors **red = **(A→G,T→C) **cyan=**(C→T,G→A) **green=**(A→T,T→A) **black=**(C→A,G→T) **blue=**(A→C,T→G) **purple=**(C→G,G→C).

We also found that the nearest-neighbor nucleotide context affects the probability of substitution errors. We therefore introduced *context-dependent* substitution probabilities into *DADA*, allowing for dependence on the nucleotides immediately preceding and following the substituted nucleotide. Such probabilities are expected to exist in reverse complementary pairs for the same reason given for the *context-independent* case (again, see Figure 5); the *lir*→*ljr* probability is expected to be similar to the $\bar{\mathrm{lir}}\to \bar{\mathrm{ljr}}$ probability where $\bar{\mathrm{lir}}$ denotes the reverse complement of *lir*. The degree of symmetry in the inferred probabilities, both context-independent and context-dependent, are shown in Figure 6 for all three data sets.

The magnitude of context-dependence for these data was moderate (most context-dependent probabilities differed by <50*%* from context-independent ones) as seen in the spread of points along the diagonal in Figure 6*d*,*e*,*f *. As a result, maintaining separate probabilities for different contexts did not affect the inferred sample genotypes. Nonetheless, that *DADA* was robust to significant variation in its parameters is a strong check on the stability of its sample inference.

We have worked with data for which context-dependence is large and has a strong effect on clustering. Therefore, we leave use of context-dependence as an optional feature of *DADA*, either as a consistency check, or when justified by the amount and nature of the data. But a caution is in order: with modest sized data sets, or if the sequences are too similar, use of the context-dependent rates could result in over-fitting and calling too many errors. However if this did occur, the expected complementarity symmetry of the inferred error probabilities would be unlikely to obtain unless the sequences were read in both directions.

## Discussion

*DADA* explicitly incorporates read abundance when deciding whether sequences are genuine or errors; if there are many identical reads of a sequence, *DADA* will be more likely to infer an underlying sample genotype, even if individually those reads would be consistent with being an error from a nearby genotype. Furthermore, *DADA* implicitly assumes, via the error model, that reads near highly abundant sequences are far more likely to be errors. In contrast, previous methods have typically treated each read independently. *AmpliconNoise* partially incorporates abundance by weighting the prior probability that a read belongs to a cluster by the frequency of that cluster, but this is weaker than *DADA*, where dependence on cluster size shows up in a binomial coefficient (see Methods), especially for high-abundance errors. By using both sequence identity and abundance in this way, *DADA* is able to disentangle real diversity from errors at finer scales than previous methods, even when tuned to be very conservative.

However, full incorporation of abundance information makes *DADA* sensitive to early-stage PCR effects and the mis-estimation of error probabilities. The problem of early-stage effects is particularly pronounced in these data: when clustered with *Ω*_*a*_=*Ω*_*r*_=10^−3^, the *Artificial* data produces 68 false positives (we would have expected no false positives if the model assumptions were not violated). The majority of these sequences have 2-5 reads and 2-4 errors. Such problems would be typical of moderately *oversampled* PCR, the regime in which initial sample molecules are typically sampled multiple times, allowing a single error during early stages to show up in more than one read.

In lieu of an abundance statistic that appropriately compensates for this affect, we deal with this problem by lowering the sensitivity of the algorithm by tuning down *Ω*_*a*_. Further, because the probability given to each sequence scales as the error probabilities to the power of the number of reads (see Methods), if certain error parameters are larger than estimated in certain contexts, then the statistical significance of an error with many reads can be substantially overestimated. This problem gets progressively worse for deeper data sets, as all one-away errors begin to take on many reads. In anticipation of this problem, we have introduced nearest-neighbor context-dependence of error rates (see Methods). These had no impact on the final clustering for the test data presented, but in other data sets with larger context-dependent effects, we found a reduction in diversity estimates when context-dependence was included (data not shown).

*DADA* is a *divisive* hierarchical clustering algorithm: all sequences are assigned to a single cluster that is subdivided until the clustering fits an error model. Previous methods, including *AmpliconNoise* and simple OTU-clustering, have predominantly taken the opposite, *agglomerative* approach, which starts with too many clusters and merges them until some condition is met. This gives *DADA* a practical advantage, as the computational and space requirements (especially the number of alignments to perform and store) scale with the square of the initial number of clusters [20]. The typical problem of divisive methods – that the number of possible splittings is too large – is handled in *DADA* by seeding new clusters with sequences that are identified as not being errors and allowing other sequences, e.g. errors associated with the new clusters, to relocate if they become more probable by doing so.

Finally, *DADA* uses unsupervised learning to acquire error probabilities from the data that it is given. As PCR protocols vary in their choice of polymerase and number of rounds, these parameters vary by data set, perhaps greatly. This makes the universality of *DADA*’s approach especially attractive, and will be important as new sequencing methods come into use such as longer read-length and paired-end *Illumina* that commonly make substitution as well as indel errors [21]. While it now relies on training data to establish error parameters, *AmpliconNoise* could be embedded in the same procedure of estimating error probabilities after each successive round of clustering, but this would multiply the computation requirements by a factor of the number of rounds of re-estimation, compounding the problem of its slower speed.

## Conclusions

OTUs serve as a rough analogue for microbes of the more clearly defined taxonomic groups of higher organisms. However, the repurposing of the OTU concept to the problem of inferring sample genotypes from error-prone metagenomic sequence data has serious and inherent shortcomings. The absence of an error model causes estimates of diversity, especially species richness, to depend strongly on experimental variables such as the size of the data set, the length of the region sequenced, and the details of the PCR/sequencing chemistry. These shortcomings are not amenable to simple fixes; it is not possible to separate real diversity from errors using an OTU approach when the diversity and the errors exist at similar scales (as measured by Hamming distance), as is the case in many metagenomic studies. *PyroNoise* and *AmpliconNoise* have demonstrated the usefulness of denoising sequence data with statistical, physically-based error models. These methods are based on the classical statistical technique of expectation-maximization. We have presented an alternate approach, *DADA*, which is more targeted to the particular task of producing conservative estimates of diversity from noisy sequence data. It is much faster and more capable of resolving fine-scale diversity while maintaining a lower false positive rate.

We did not achieve our goal of complete freedom from ad hoc parameters in this work. Even though *Ω*_*a*_, our input parameter, has a simple probabilistic meaning that is data set independent, there are corrections to our PCR model, and as a result *Ω*_*a*_ takes on an ad hoc quality in this analysis. Nonetheless, *Ω*_*a*_can be coarsely tuned from the data itself in the way shown. Alternatively, for conservative diversity estimates, *Ω*_*a*_ may be set to very small values (such as 10^−100^), and the resolution of the algorithm may be directly quantified. *DADA* not only guesses what is there, but knows what would have been missed if it were present, making *Ω*_*a*_ ad hoc but not arbitrary.

Much work remains to be done, and it is not yet clear how the algorithms will fare with extremely rich fine-scale diversity as occurs for the antibody repertoire of B-cells and T-cells of the human immune system [22,23]. *DADA* must be equipped with statistics that correctly describe the abundance distribution of sequencing errors when a realistic model of PCR is used in which some reads are the result of shared lineages. More sophisticated methods for chimera detection that explicitly parameterize the chimera formation process analogously to the substitution and indel processes are also needed. Finally, these methods must be fully adapted and tested on sequencing platforms other than Roche’s *454*.

## Methods

### General notation

From a sequencing data set $S=\{s_{x},\;r_{x}\}$, where *r*_*x*_ is the number of individual reads of each distinct sequence *s*_*x*_, we would like to construct an estimate $G=\{G_{\alpha }\}$ of the set of genotypes in the sample that gave rise to S. With this aim, we construct a partition B of the sequences {*s*_*x*_} where each $B_{\alpha }\in B$ is a collection of sequences hypothesized to have originated from a common *G*_*α*_, and notate the number of reads assigned to *G*_*α*_by $\rho_{\alpha }=\underset{x|s_{x}\in B_{\alpha }}{\sum }r_{x}$. Because each *s*_*x*_ can reside in only one *B*_*α*_ and it is assumed that *G*_*α*_ is the source of all *s*_*x*_ in *B*_*α*_, this framework does not allow for multiple *G*_*α*_ to contribute reads to the same *s*_*x*_. Allowing the latter is likely to affect G only in special cases and adds complications.

### Treatment of insertions and deletions: the construction of *indel families*

In addition to substitution errors, reads acquire insertions and deletions (indels) during amplification and sequencing. Both substitutions and indels could be used to parameterize an error model, but here we focus on substitutions and do not attempt to characterize the statistics of indels. Instead, we collapse together all the reads of sequences within each *B*_*α*_that differ from each other only by the location of indels in their alignments to *G*_*α*_, forming subsets of each *B*_*α*_ that we call *indel families*. We call the indel families $F(S,B,G)=\{s_{y},r_{y}\}$, where each **s**_*y*_ refers either to a subset of some *B*_*α*_ or the sequence identical with *G*_*α*_ except for the substitution errors of its constituents, and **r**_*y*_ is the number of reads in the family. The **r**_*y*_ of each indel family will be used to test whether B agrees with an error model, i.e. whether the substitution errors observed on the families in each *B*_*α*_ was not too improbable under an error model of substitution errors.

Alignments between sequences and each *G*_*α*_ in this paper took place with a scoring matrix of 5 + log*T*(to make them comparable with NCBI BLAST’s NUC.4.4 matrix [24]), where *T*, introduced below, is a matrix of substitution error probabilities. We used a gap penalty of −4 and a hompolymer gap penalty of −1. The gap penalty had to be less than half the smallest mismatch score or alignments would favor a pair of indels to that mismatch. The worst mismatch score tended to be about −6, and so −4 was chosen as a gap penalty to allow as many gaps as possible without making any mismatches prohibited within alignments.

### The independence between substitution errors on different reads implies a binomial distribution for the number of reads of each family

If the occurrence of substitution errors on different reads are independent events, then each read of genotype *G*_*α*_ has an i.i.d. one-trial multinomial distribution with parameters Λ={*λ*_*yα*_}, which we call the *genotype error probabilities*, to belong to each indel family **s**_*y*_. Λ also parameterizes the probability distribution for *R*_*y*_, the number of reads of family *y*: if **s**_*y*_⊆*B*_*α*_, then because *R*_*y*_ is the sum of *ρ*_*α*_ Bernoulli random variables each with success probability *λ*_*yα*_, it follows the binomial distribution, *R*_*y*_∼*Bin*(*λ*_*yα*_,*ρ*_*α*_). The assumption of independence between reads does not hold if early round PCR errors may be sampled multiple times in the final sequence data. Then, if we condition on having observed a particular error on some other read, the probability to observe it additional times is increased.

### Λ may be constructed from simple nucleotide transition matrices

If the occurrence of substitution errors on different sites of the same read are independent events that do not depend upon the absolute position of the sites, then we can write each *λ*_*yα*_ in terms of a homogeneous Markov chain *T*, whose elements we call *nucleotide error probabilities*. The simplest useful model of this sort is the 4×4 transition matrix *T*_*ij*_=*P*(*j*|*i*) with, for example *P*(C|A) the probability for taking nucleotide A in the sample to C in the data (*i,j* will always index nucleotides), and this is the model used by *AmpliconNoise*. These probabilities generate the genotype error probabilities Λ via

(1) $$\lambda_{\mathrm{y\alpha}}=\underset{n}{\prod }T_{\alpha_{n}y_{n}}$$

where *α*_*n*_ and *y*_*n*_ denote the *n*^*th*^ nucleotides of *G*_*α*_ and **s**_*y*_ (also let *λ*_*xα*_=*λ*_*yα*_ for all *x*|*s*_*x*_∈**s**_*y*_, used in Algorithm Algorithm 1).

If the nucleotide error probabilities at each site can depend upon the nearest-neighbor flanking nucleotides, we can keep track of a transition matrix *T*^(*L*,*R*)^ for each possible (*L,R*) pair of flanking nucleotides such that $T_{\mathrm{ij}}^{\left(L,R\right)}=P(\mathrm{LjR}|\mathrm{LiR})$, with *P*(ATC|AGC) the error probability for taking AGC to ATC. This generalization, which we call *context-dependence*, increases the number of free parameters from 12 to 192: there are 16 possible pairs of flanking nucleotides each with a 12 free parameter stochastic matrix. *DADA* may be run with or without context-dependence, but due to the risk of overfitting and because we saw no substantial effect on the outcome of clustering when it was used, context-dependence was not used to produce the results presented in this paper.

### Assessing fit with an error model via tail probabilities

In order to assess whether G and B fit the error model Λ, we introduce two statistics, *p*_*y*_and *q*_*α*_: *p*_*y*_ is the probability of having seen at least **r**_*y*_reads of **s**_*y*_ given that we saw at least one and *q*_*α*_is the probability of having seen at least one read with a genotype error probability at least as small as the smallest genotype error probability of an observed indel family in *B*_*α*_, $\lambda_{\alpha }^{*}=\underset{y|s_{y}\subseteq B_{\alpha }}{\min}\lambda_{\mathrm{y\alpha}}$.

#### *p*_*y*_: *the abundance p-value*

Call $R_{y}^{+}$ the number of reads of **s**_*y*_given that we observed at least one:

$$\begin{array}{lll} P(R_{y}^{+}=r) & =P(R_{y}=r|r>0)\; & \;\\ & =\frac{P(R_{y}=r)}{1-P(R_{y}=0)}∼\frac{\text{Bin}(\lambda_{\mathrm{y\alpha}},\rho_{\alpha })}{1-{\left(1-\lambda_{\mathrm{y\alpha}}\right)}^{\rho_{\alpha }}}\; & \; \end{array}$$

Given the definition of *p*_*y*_above,

py=P(Ry+≥ry)=∑r=ry∞P(Ry+=r)=∑r=ry∞ραrλyαr1−λyαρα−r1−1−λyαρα

We refer to this as the *abundance p-value* because it evaluates the probability of having observed more extreme abundances under the null hypothesis that each **r**_*y*_was generated by the error model. Because one abundance p-value is generated for each indel family, we use a Bonferroni correction and compare each *p*_*y*_with $\Omega_{a}/|F|$, where *Ω*_*a*_ is a joint significance threshold that is provided to *DADA* by the user.

If we had not conditioned on having observed at least one read of each family, then the *unobserved* families would not have born any significance (they would all have *p*_*y*_=1), but before looking at the data these families *could* have been significant. This would create a difficulty in choosing an appropriate multiple hypothesis correction; a naive Bonferroni correction of $\underset{\alpha }{\prod }4^{L_{\alpha }}$ with *L*_*α*_ the length of *G*_*α*_, which treats all possible families as tested hypotheses, would deprive the p-value of any statistical power. Conditioning on *R*_*y*_>0 and evaluating only the observed sequences avoids this complication. However, any family with **r**_*y*_=1 obtains *p*_*y*_=1 regardless of the smallness of *λ*_*yα*_, which necessitates our second statistic, *q*_*α*_.

#### *q*_*α*_: *the read p-value*

For each cluster *B*_*α*_, we compute the probability *q*_*α*_, which we call the *read p-value*, that there is at least one read with a genotype error probability at least as small as $\lambda_{\alpha }^{*}$. Let *l*_*α*_be a random variable representing the smallest genotype error probability when *ρ*_*α*_ reads of *G*_*α*_ are generated according to Λ. Then

$$q_{\alpha }=P(l_{\alpha }\leq \lambda_{\alpha }^{*})=1-{\left(\underset{e|\lambda_{\mathrm{e\alpha}}>\lambda_{\alpha }^{*}}{\sum }\lambda_{\mathrm{e\alpha}}\right)}^{\rho_{\alpha }}$$

where *e* iterates over all $4^{L_{\alpha }}$ sequences, and *λ*_*eα*_are the genotype error probabilities of these sequences. Evaluating the sum in this form would be computationally wasteful; instead we iterate over sets of sequences that share the same types of substitution errors. We index these sets by 4×4 off-diagonal matrices *γ* whose elements *γ*_*i*≠*j*_specify the number of i’s on a genotype that appear as j’s on the sequence. The genotype error probability for sequences of type *γ* away from genotype *G*_*α*_is *λ*_0*α*_×*λ*_*γ*_ with $\lambda_{0\alpha }=\underset{i}{\prod }T_{\mathrm{ii}}^{n_{\mathrm{\alpha i}}}$ the probability of having no errors (where *n*_*αi*_ is the number of nucleotides of type *i* on *G*_*α*_) and $\lambda_{\gamma }=\underset{i,j}{\prod }{\left(\frac{T_{\mathrm{ij}}}{T_{\mathrm{ii}}}\right)}^{\gamma_{\mathrm{ij}}}$, which is independent of *α*. We also need the number of distinct sequences of type *γ*for each *G*_*α*_, which we call the *degeneracym*_*γ*_(*α*) of *γ* on *G*_*α*_. This is computed by taking a product over multinomial coefficients: $m_{\gamma }(\alpha )=\underset{i}{\prod }\frac{n_{\mathrm{\alpha i}}!}{(n_{\mathrm{\alpha i}}-\underset{j}{\sum }\gamma_{\mathrm{ij}})!\underset{j}{\prod }\gamma_{\mathrm{ij}}!}$. Rewritten as a sum over *γ*, *q*_*α*_becomes

$$q_{\alpha }=1-{\left(\lambda_{0\alpha }\underset{\gamma |\lambda_{\gamma }>\lambda_{\alpha }^{*}/\lambda_{0\alpha }}{\sum }m_{\gamma }(\alpha )\lambda_{\gamma }\right)}^{\rho_{\alpha }}$$

Vectors of *λ*_*γ*_ and *m*_*γ*_(*α*) can be computed starting with *γ*representing the more common errors and extended to more rare errors as needed to compute p-values for smaller $\lambda_{\alpha }^{*}$. Finally, rather than maintaining vectors of *m*_*γ*_(*α*) for each *α*, we keep one for each of a small number of possible base compositions and interpolate between the *q*_*α*_that would result from each of these in order to approximate the *q*_*α*_ that would result from the exact base composition of *G*_*α*_. Because one *q*_*α*_ is generated for each *B*_*α*_, the *q*_*α*_ are then compared with $\Omega_{r}/|B|$, where *Ω*_*r*_ is another joint significance threshold provided to *DADA* by the user, in order to determine whether any $\lambda_{\alpha }^{*}$ are too small to be the result of errors.

### Maximum likelihood estimate (mle) of error probabilities

After forming a partition B of S that fits the error model generated by *T*, *DADA* updates *T* to its maximum likelihood estimate given this partition. The likelihood of *T* given S and B is

$$ℒ(T|S,B)=\underset{\alpha }{\prod }\underset{x|s_{x}\in B_{\alpha }}{\prod }\underset{n}{\prod }T_{\alpha_{n}x_{n}}^{r_{x}}$$

where *α*_*n*_ and *y*_*n*_ denote the *n*^*th*^ aligned nucleotides of *G*_*α*_and *s*_*x*_. For the case without context-dependence, the likelihood may be rewritten as

$$ℒ(T|S,B)=\underset{i}{\prod }\left(\underset{j\neq i}{\prod }T_{\mathrm{ij}}^{N_{\mathrm{ij}}}\right){\left(1-\underset{j\neq i}{\sum }T_{\mathrm{ij}}\right)}^{N_{\mathrm{ii}}}$$

where *N*_*ij*_ is the total number of ***js*** in S that result from *is* in G according to B. The maximum likelihood equations for the off-diagonal elements of *T*, $\frac{\partial ℒ(T|S,B)}{\partial T_{i\neq j}}(\{{\hat{T}}_{\mathrm{mle},\mathrm{ij}}\})=0$, are solved by maximum likelihood estimate

$${\hat{T}}_{\mathrm{mle},i\neq j}=\frac{N_{\mathrm{ij}}}{N_{i}}$$

where *N*_*i*_=∑_*j*_*N*_*ij*_ (the diagonal ${\hat{T}}_{\mathrm{mle},\mathrm{ii}}=1-\underset{j\neq i}{\sum }{\hat{T}}_{\mathrm{mle},\mathrm{ij}}$ are set by normalization). Analogously, for the *context-dependent* case, the mle estimate is

$${\hat{T}}_{\mathrm{mle},\mathrm{ij}}^{\left(L,R\right)}=\frac{N_{\mathrm{LijR}}}{N_{\mathrm{LiR}}}$$

where *i*≠*j*, *L,R* are any pair of left and right flanking nucleotides, *N*_*LijR*_ is the total number of *LjR* codons in S that result from *LiR* codons in G according to B, and *N*_*LiR*_ = ∑_*j*_*N*_*LijR*_.

### Algorithm for inferring the sample genotypes and error probabilities

The p-values *p*_*y*_ and *q*_*α*_ are the basis for an algorithm that alternatively updates B and the nucleotide error probabilities *T*, which may be specified by the user as either context-independent or dependent, denoting their values after *t* iterations by $B^{T}$ and *T*^*t*^, in order to improve the likelihood of the data. This is similar to the *hard-EM* algorithm except that the partition $B^{T}$ at each step is the result of a model-based divisive hierarchical clustering approach and does not maximize the probability of S given *T*^*t*^. The algorithm requires two user inputs, *Ω*_*a*_and *Ω*_*r*_, which are the joint significance thresholds for the abundance and read p-values.

### Algorithm 1

*DADA* Sequence clustering algorithm [15]

*T*^0^= ${\hat{T}}_{\mathrm{mle}}(B_{0})$, where $B_{0}$ is the trivial partition containing the entire S.

*t*←1

repeat

$B^{T}\leftarrow B_{0}$

repeat

**if**$B^{T}\neq B_{0}$

 start a new cluster within $B^{T}$ containing the most statistically inconsistent family.

repeat

 update $\left\{{Ĝ}_{\alpha }\right\}$

$\rho_{\alpha }\leftarrow \underset{x|s_{x}\in B_{\alpha }}{\sum }r_{x}$

$\lambda_{\mathrm{x\alpha}}=\underset{n}{\prod }T_{\alpha_{n}x_{n}}$ (or $T_{\alpha_{n}x_{n}}^{\left(\alpha_{n-1},\alpha_{n+1}\right)}$ if context-dependence is on)

 each *s*_*x*_joins *B*_*α*_where $\alpha ={\text{arg max}}_{\alpha^{\prime \prime }}(\rho_{\alpha^{\prime \prime }}\lambda_{x\alpha^{\prime \prime }})$

**until**$B^{T}$ is unchanged

 update {*p*_*y*_} and {*q*_*α*_}

**until**$\min p_{y}\geq \Omega_{a}/|F|$ and $\min q_{\alpha }\geq \Omega_{r}/|B|$

$T^{T+1}={\hat{T}}_{\mathrm{mle}}(B^{T})$

*t*←*t* + 1

**until*T*** has converged

There are three levels of nesting, each beginning with a **repeat** statement in Algorithm Algorithm 1. From outer to inner, we give a qualitative description of their purpose: 

1. Starting with *T*^0^, the maximum likelihood nucleotide error probabilities given the trivial partition $B_{0}$ of all sequences into a single cluster, the outermost loop iteratively updates B and ***T*** until *T* converges. We have observed cases where *T* does not completely settle down but fluctuates within a small basin of attraction. To deal with such cases, *DADA* terminates if *T* ever returns to a previously held value or ||*T*^*t*^−*T*^*t*−1^|| <*∊*, where the tolerance *∊* = 1*e* − 9 is used as a default and may be altered by the user. If convergence has not been reached in ten rounds, *DADA* terminates with a warning message.

2. For each *T*^*t*^, the next loop begins with the trivial partition, $B^{T}=B_{0}$, and adds blocks to $B^{T}$ until the {*p*_*y*_} and {*q*_*α*_} do not allow rejection of the error model at joint significance levels *Ω*_*a*_and *Ω*_*s*_. New $B_{\alpha }^{T}$ are seeded by the sequences in families with the smallest p-values. If statistically significant families exist under both p-values, then those significant under the abundance p-value take priority for starting new clusters. This approach avoids the need to put an explicit penalty on the number of blocks of B, instead aiming for the smallest B under which the current error model cannot be rejected.

3. After adding a new block $B_{\alpha }^{T}$ to $B^{T}$, the innermost loop raises the probability of the data by reassigning each sequence to the block that would produce (under the error model) the largest expected number of reads of that sequence. The putative genotype of a cluster, *G*_*α*_, is also updated if a cluster *B*_*α*_has a new consensus sequence. This continues until sequences cease changing clusters.

### Appendix 1: chimeras, contaminants, and missing or incorrect Sanger sequences

There are disagreements between the Sanger sequences of the clonal isolates used to construct the data sets and the denoised sequences of *DADA* and *AmpliconNoise* that are due to sources other than PCR substitutions and pyrosequencing errors. These include contamination, chimeric sequences that result from the co-amplification of genomes with regions over which they exactly match, Sanger errors, the absence of any reads of two sample genotypes, and disagreements between the Sanger sequences and majority of the *454* reads about the lengths of several homopolymers (for example, not a single *454* read matched four of the Sanger sequences while many were identical except for the presence of a single deletion on a G homopolymer). In order to evaluate the relative performance of *DADA* and *AmpliconNoise* as denoising algorithms, it was necessary to identify which disagreements between Sanger and denoised sequences were due to these sources and which, falling outside these categories, were due to algorithmic shortcomings. We chose criteria for classifying errors of these types and applied them to the sequences denoised by *DADA* and *AmpliconNoise* (Table 3).

**Table 3:** Additional sources of noise and false positives

	**DADA**					**AmpliconNoise**				
**Sample**	**Denoised**	**Clone**	**Chim**	**Contam**	**Other**	**Denoised**	**Clone**	**Chim**	**Contam**	**Other**
Divergent	43	23	18	2	0	51	23	23	3	2
Artificial	65	50	14	0	1	73	44	21	0	8
Titanium (s10)	274	80	185	3	6	163	71	82	2	8
Titanium (s25)						304	76	203	2	23

For each data set and both algorithms: the total number of denoised sequences, the number that matched one of the Sanger sequenced clones, the number classified as chimeras, the number classified as contaminants, and all other false positives.

We began by correcting possible errors in the Sanger sequences. In the *Divergent* and *Artificial* data sets there were disagreements between very high abundance denoised sequences and their nearest neighbor Sanger sequences (12/23 *Divergent* Sanger sequences and 63/90 *Artificial* Sanger sequences). The denoised sequences were a consensus of thousands of pyrosequencing reads and did not differ from the Sanger sequences near homopolymers; rather, all disagreements were non-homopolymer related deletions near the starts of the reads. It was confirmed (Chris Quince, personal communication) that all bases of Sanger sequences aligning to sites within 13 nucleotides (nts) of the forward primer of the pyrosequencing reads had been removed in *Q11*, and so were likewise removed in all our analysis. In the *Titanium* data, the denoised sequences closest to eight of the Sanger sequences had over 100 reads but differed from them by one or two homopolymer deletions at several long homopolymers. *DADA* and *AmpliconNoise* (s10 and s25) agreed on the presence of the deletions in all of these sample genotypes, and there were more copies of the error-containing sequences than the Sanger sequences in the raw data (Table 4), suggesting either an error probability greater than 50% for the combined amplification/pyrosequencing process or problems with the Sanger sequences. Therefore, we did not consider these disagreements to be false positives or false negatives for either algorithm.

**Table 4:** *Titanium* genotypes with more error-containing than Sanger sequence matching reads

**Reads of nearby pyrosequence**	**Reads of sanger sequence**	**Errors**
5	0	G8→G7 (301),
		G5→G4 (354)
70	0	G6→G5 (133)
75	1	C7→C6 (313)
21	18	G6→G5 (133)
14	0	G6→G5 (133)
80	0	G6→G5 (133)
77	3	G6→G5 (133)
80	1	G6→G5 (133)

The difference between the Sanger and *454* sequences and their locations are given. For example, G6→G5 (133) means that a homopolymer beginning at the 133^*rd*^base with 6 Gs in a Sanger sequence showed up as 5 Gs in most of the pyrosequence reads. All disagreements consist of deletions in the *454* reads on long G/C homopolymers relative to the Sanger sequences, and the majority of the disagreements occurred on one locus, site 133.

Next we identified chimeras: sequences consisting of two sections with one section a close match to one sample genotype and the other a close match to a second sample genotype. These can be produced in substantial quantities by PCR [25]. Analogously to *Q11*, for each denoised sequence we computed the Hamming distance to the nearest Sanger sequence and to the nearest exact chimera by considering all possible breakpoints between all pairs of sequences of higher abundance (a chimera will have fewer reads than its parents unless it acquires substantial PCR bias). For a denoised sequence to be classified as a chimera, we required that it be at least 3 nts closer to the nearest exact chimera than the nearest sample genotype and within 5 nts of the optimal chimera (also analogous to the procedure used in *Q11*). We waived the 3 nt improvement criteria for denoised sequences that were identical to exact chimeras, which occurred for some particularly highly abundant chimeras between closely related sample genotypes. All data sets had a large number of chimeras amongst their denoised reads, with *Titanium* having more chimeras than sample genotypes (both algorithms), highlighting how essential accurate chimera identification is in tandem with the correction of PCR and sequencing errors.

Finally, we found several sequences too far from any sample genotypes or exact chimeras to be explained by being errors away from either. Some of these sequences were similar to previously observed sequences found on *GenBank* (Table 5). We classified as a contaminant any sequence within 2 nts of a *GenBank* sequence and at least 5 nts closer to a GenBank sequence than any sample genotype or chimera. We found a mixture of contaminants likely to come from the original sample (lake water bacteria), and contaminants that may have entered the sample during processing and sequencing (bacteria previously observed in human skin, a human mouth, and soil samples). These contaminants were not previously mentioned in *Q11* but were straightforward to detect when looking at *DADA*’s denoised sequences, in part because having a smaller pool of algorithmic false positives makes identifying contaminants much easier.

**Table 5:** Contaminants

**Accession**	**Reads/Frequency**	***D*_*GB*_**	***D*_*sample*_**	***D*_*chim*_**	**Source**	**Type**	**DADA**	**AN**
Divergent								
FR697039	14/4×10^−4^	0	11	10	Lake Water	Bacterium	Y	Y
EU633742	1/3×10^−5^	1	9	9	Showerhead	Methylobacter	Y	Y
JF515955	1/3×10^−5^	1	8	8	Soil	Nitrosomonadaceae	N	Y
Titanium								
FJ004768	77/3×10^−3^	2	39	27	Soil	Bacterium	Y	Y
JF190756	1/4×10^−5^	1	40	27	Human Skin	Bacterium	Y	Y
JQ462329	2/8×10^−5^	0	7	5	Human Mouth	Bacterium	Y	N

*D*_*GB*_, *D*_*sample*_and *D*_*chim*_are the Hamming distances to the given GenBank entry, the nearest sample genotype, and the optimal chimera for each putative contaminant denoised sequence. No entries are given for the *Artificial* data set because no likely contaminants were found in the denoised sequences of either algorithm. The last two columns show which contaminants were present in the denoised sequences of each algorithm (“Y” if found and “N” if not found).

In classifying false negatives, we sought to evaluate the ability of the algorithms to detect the presence of genuine diversity in the pyrosequencing reads. However, not all clones used to construct the samples in *Q11* had exact matches amongst the pyrosequencing reads: one Sanger sequence in the *Artificial* data set (#69 in *Q11*) was 29 nts away from the nearest *454* read and one Sanger sequence in the *Titanium* data set (#66 in *Q11*) was 61 nts away from the nearest *454* read. We assumed that these were missing from the *454* data, and they do not contribute to the false negatives of either algorithm. Further, a number of clones were identical to each other up to the point of truncation of the pyrosequencing reads. Finally, a number of the *Titanium* clones differed from each other only by the presence of Ns, bases that Sanger was unable to resolve. In such cases, we collapsed clones together and assumed the non-N containing Sanger sequence was correct. Table 6 gives the number of distinct (up to Ns) clones that are present in the data, and how many had been used to construct the sample.

**Table 6:** Detectable genotypes

**Sample**	**Genotypes**	**Present and distinct**
Divergent	23	23
Artificial	90	50
Titanium	91	80

The number of genotypes used to construct the sample and the number that were present and distinct and so could detected by a denoising algorithm.

Several aspects of this *post-processing* pipeline – especially contaminant identification– utilize knowledge of the sample genotypes and do not constitute useful methods for non-test data. Our approach to chimera identification does not utilize sample genotype information, but requires more development to be applied to non-test data: it does not search for higher-order chimeras that are combinations of three or more parental sequences, the criteria for being labelled as a chimera do not scale with error rates and read lengths, and no attempt has been made to realistically model the chimera formation process. Our goal has been only the isolation of errors due to PCR and sequencing error.

### Appendix 2: *Ω*_*a*_robustness

To assess whether *DADA*’s performance in this paper was the result of the fine-tuning of *Ω*_*a*_, we evaluated each data set under all three *Ω*_*a*_values. The results are given in Table 7 and demonstrate that the same results would have been achieved by using *Ω*_*a*_ = 10^−100^ for all three data sets.

**Table 7:** False positives and false negatives for each data set with *Ω*_*a*_ = {10^−15^,10^−40^,10^−100^} and *Ω*_*r*_ = 10^−3^

					**Divergent**		**Artificial**		**Titanium**
				***Ω*_*a*_**	**False Pos**	**False Neg**	**False Pos**	**False Neg**	**False Pos**	**False Neg**
				10^−15^	0	0	10	2	7	0
				10^−40^	0	0	1	2	6	0
				10^−100^	0	0	1	2	6	0

## Competing interests

The authors declare that they have no competing interests.

## Authors’ contributions

MJR designed the algorithm, wrote the software, performed the analyses, and wrote the paper. BJC, DSF, and SPH provided assistance and advice with algorithm design, comparative analysis, and the paper. All authors read and approved the final manuscript.
